# Natural Nanoparticles for Drug Delivery: Proteomic Insights and Anticancer Potential of Doxorubicin-Loaded Avocado Exosomes

**DOI:** 10.3390/ph18060844

**Published:** 2025-06-04

**Authors:** Dina Salem, Shaimaa Abdel-Ghany, Eman Mohamed, Nada F. Alahmady, Amany Alqosaibi, Ibtesam S. Al-Dhuayan, Mashael Mashal Alnamshan, Rebekka Arneth, Borros Arneth, Hussein Sabit

**Affiliations:** 1 Department of Agriculture Biotechnology, College of Biotechnology, Misr University for Science and Technology, P.O. Box 77, Giza 12566, Egypt; dina.salim@must.edu.eg; 2 Department of Environmental Biotechnology, College of Biotechnology, Misr University for Science and Technology, P.O. Box 77, Giza 12566, Egypt; shaimaa.ibraheem@must.edu.eg; 3 Department of Pharmaceutical Biotechnology, College of Biotechnology, Misr University for Science and Technology, P.O. Box 77, Giza 12566, Egypt; emann90884@student.must.edu.eg; 4 Department of Biology, College of Science, Imam Abdulrahman bin Faisal University, P.O. Box 1982, Dammam 31441, Saudi Arabia; nalahmadi@iau.edu.sa (N.F.A.); amgosaibi@iau.edu.sa (A.A.); ialdhuayan@iau.edu.sa (I.S.A.-D.); malnamshan@iau.edu.sa (M.M.A.); 5 Clinic of Internal Medicine, Hospital of the Universities of Giessen and Marburg (UKGM), Justus Liebig University Giessen, Feulgenstr. 12, 35392 Giessen, Germany; rebekka.arneth@innere.med.uni-giessen.de; 6 Institute of Laboratory Medicine and Pathobiochemistry, Molecular Diagnostics, Hospital of the Universities of Giessen and Marburg (UKGM), Philipps University Marburg, Baldingerstr. 1, 35043 Marburg, Germany; 7 Institute of Laboratory Medicine and Pathobiochemistry, Molecular Diagnostics, Hospital of the Universities of Giessen and Marburg (UKGM), Justus Liebig University Giessen, Feulgenstr. 12, 35392 Giessen, Germany; 8 Department of Medical Biotechnology, College of Biotechnology, Misr University for Science and Technology, P.O. Box 77, Giza 12566, Egypt

**Keywords:** exosomes, drug delivery, plant-derived exosomes, doxorubicin, anticancer therapy

## Abstract

**Background**: Exosomes have recently attracted significant attention for their potential in drug delivery. Plant-derived exosomes, in particular, may serve as direct anticancer agents due to their unique characteristics, including immunogenicity, biocompatibility, safety, cell-free nature, and nanoscale structure. **Methods**: This study characterizes *Persea americana* (avocado)-derived exosomes, exploring their anticancer properties, proteomic profile, and therapeutic potential. **Results**: Isolated exosomes exhibited a diameter of 99.58 ± 5.09 nm (non-loaded) and 151.2 ± 6.36 nm (doxorubicin (DOX)-loaded), with zeta potentials of −17 mV and −28 mV, respectively. Proteomic analysis identified 47 proteins, including conserved exosome markers (GAPDH, tubulin) and stress-response proteins (defensin, endochitinase). Functional enrichment revealed roles in photosynthesis, glycolysis, ATP synthesis, and transmembrane transport, supported by protein–protein interaction networks highlighting energy metabolism and cellular trafficking. DOX encapsulation efficiency was 18%, with sustained release (44.4% at 24 h). In vitro assays demonstrated reduced viability in breast cancer (MCF-7, T47D, 4T1) and endothelial (C166) cells, enhanced synergistically by DOX (Av+DOX). Gene expression analysis revealed cell-specific modulation: Av+DOX upregulated *TP53* and *STAT* in T47D but suppressed both in 4T1/C166, suggesting context-dependent mechanisms. **Conclusions**: These findings underscore avocado exosomes as promising nanovehicles for drug delivery, combining biocompatibility, metabolic functionality, and tunable cytotoxicity. Their plant-derived origin offers a scalable, low-cost alternative to mammalian exosomes, with potential applications in oncology and targeted therapy. Further optimization of loading efficiency and in vivo validation are warranted to advance translational prospects.

## 1. Introduction

In recent years, human extracellular vesicles (EVs) have garnered significant attention due to their essential roles in intercellular communication, disease pathogenesis, and therapeutic applications [1,2]. However, plant-derived EVs remain poorly studied. EVs are involved in numerous biological processes across different organisms, including plants, animals, fungi, and bacteria. Based on their size, EVs are classified into three main types: apoptotic bodies (~1000 nm), microvesicles (100–1000 nm), and exosomes (50–150 nm) [3]. These vesicles serve critical functions, such as facilitating intercellular communication, eliminating cellular waste [4], modulating immune responses [5], and presenting antigens to T-cells and B-cells to activate immune defenses [6]. Moreover, EVs play a significant role in plant–pathogen interactions, as plants and microbes produce extracellular vesicles contributing to host–pathogen communication. Exosomes are composed of lipids, proteins, microRNA (miRNA), DNA, and messenger RNA (mRNA), allowing them to act as transport vehicles that shuttle molecular cargo between cells [7,8,9]. These vesicles facilitate cell communication within the same organism and between different species, playing a key role in host–pathogen interactions [10,11].

Nanoparticles have been extensively studied for drug delivery applications to improve therapeutic efficacy at lower drug concentrations. However, synthetic nanoformulations often present challenges such as toxicity and rapid clearance by the mononuclear phagocytic system (MPS) [12,13,14]. In contrast, exosomes possess unique characteristics that make them highly efficient drug delivery systems, including excellent biocompatibility, low toxicity, and minimal immunogenicity [15]. Furthermore, exosomes offer advantages such as enhanced cellular uptake, improved stability in the gastrointestinal tract, and targeted drug delivery capabilities [16]. Compared to synthetic liposomes, exosomes demonstrate superior biocompatibility, safety, apoptotic induction, drug release kinetics, and cell–substrate interaction [17]. These properties have enabled exosomes to deliver proteins, miRNAs, mRNA, and therapeutic drugs such as doxorubicin [18] and rifampicin [19]. Exosomes have demonstrated the ability to cross the blood–brain barrier, making them promising candidates for neurological drug delivery.

Compared to mammalian exosomes, plant-derived exosomes (PEs) are easier to isolate in large quantities, exhibit superior biocompatibility [20], and offer enhanced safety as drug carriers. PEs contain functional proteins and miRNAs that modulate the expression of various human genes, thereby exerting therapeutic effects [21,22]. In addition to their nucleic acid and protein content, PEs are rich in bioactive phytochemicals, many of which exhibit potent therapeutic activities against various diseases, including cancer [23,24]. Several studies have demonstrated the anticancer potential of plant-derived exosomes, either in their free form or when loaded with chemotherapeutic agents [25].

Doxorubicin (DOX) is a widely used chemotherapeutic agent that effectively treats various malignancies, including breast cancer. Its cytotoxic effects are primarily attributed to DNA intercalation, inhibition of topoisomerase II, and the generation of free radicals, leading to oxidative damage and apoptosis in cancer cells [26]. However, DOX-induced toxicity limits its clinical use, necessitating the development of novel drug delivery systems to enhance its therapeutic efficacy while reducing adverse effects [27,28,29].

*Persea americana* (avocado) is a medicinal plant rich in diverse phytochemicals that exhibit various pharmacological effects, including antioxidant, antidiabetic, and weight-loss properties [30]. Its methanolic extract has also demonstrated significant anticancer activity against different cancer cell lines.

This study investigates the potential of *Persea americana*-derived exosomes as a natural drug delivery system for doxorubicin. Specifically, the research seeks to evaluate these exosomes’ biocompatibility, stability, and anticancer efficacy, highlighting their potential as a safer and more efficient alternative to synthetic nanocarriers for targeted cancer therapy.

## 2. Results

### 2.1. Characterization of P. americana Exosomes

**Figure 1:**
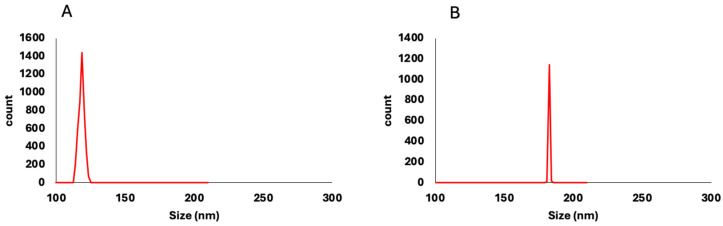
Dynamic light scattering (DLS) analysis of *P. americana*-derived exosomes (**A**) and DOX-loaded exosomes (**B**) shows a monodisperse size distribution. The peak shift in (**B**) indicates a slight increase in particle size upon doxorubicin loading. This confirms successful drug encapsulation while maintaining a uniform nanoparticle population.

**Figure 2:**
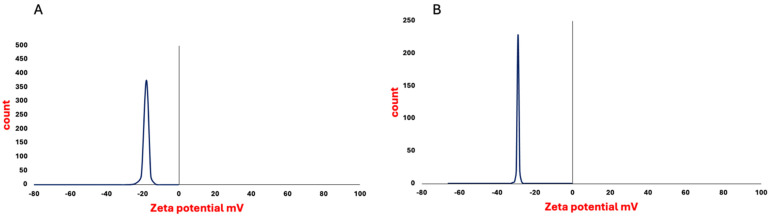
Zeta potential distribution of *P. americana*-derived exosomes (**A**) and DOX-loaded exosomes (**B**) shows a strong negative charge, enhancing colloidal stability. The high negative values suggest repulsion among particles, reducing aggregation and improving biocompatibility.

**Figure 3:**
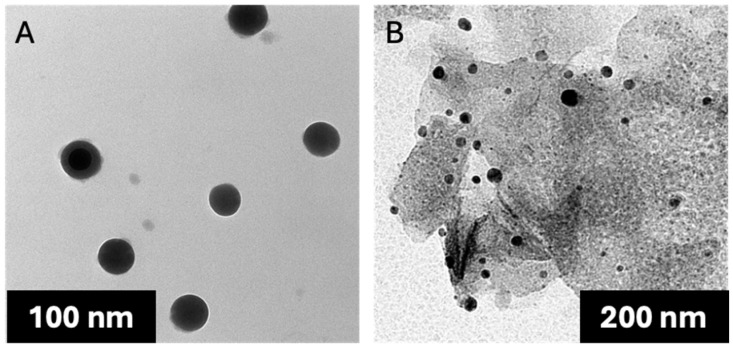
TEM image of *P. americana*-derived exosomes: (**A**) dox-loaded exosomes and (**B**) non-loaded exosomes.

The isolated, non-loaded *P. americana*-derived exosomes had a diameter ranging from 51 to 150 nm as indicated by TEM results, averaging 99.58 ± 5.09. The dynamic light scattering (DLS) results showed an average size of 119 ± 1.53 nm (Figure 1) and zeta potential of −17 mV ± 1.91 (Figure 2), while the loaded *P. americana*-derived exosomes’ diameter ranged from 126.81 to 162 nm with an average of 151.2 ± 6.36 according to TEM results (Figure 3). In comparison, the average of the DLS results was 166 ± 1.22 nm and zeta potential −28 ± 0.77 mV. As reported by previous studies, these values are located within the ranges of diameter and zeta potential of exosomes, which ranged from 30 to 150 nm [31] for diameter and −6 and −30 mV for zeta potential values. The size distribution recorded in other plant exosomes including *Citrus limon* L. fruit (50–70 nm), tomato root (50–100 nm) [32], and *Drynariae rhizoma* root-derived extracellular vesicles (40–100 nm) have larger diameter [33].

### 2.2. Proteomics of P. americana Exosome

**Figure 4:**
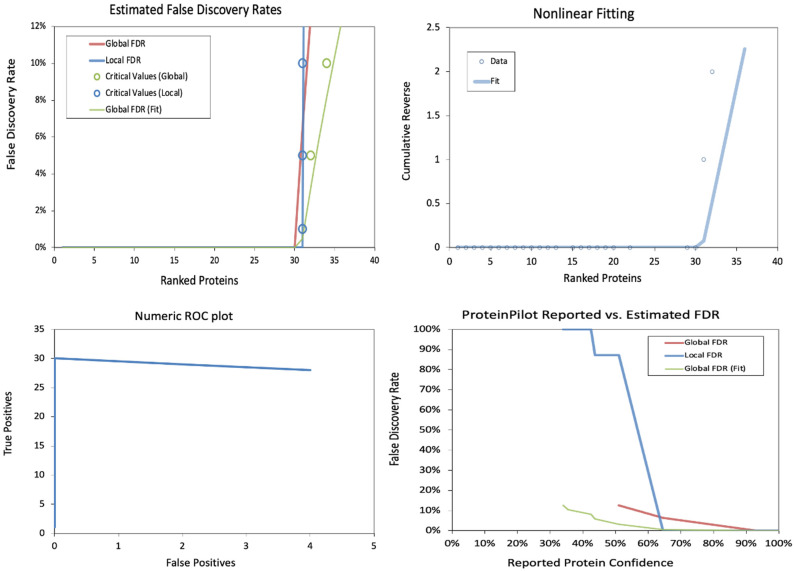
False discovery rate (FDR) analysis of identified proteins. (**Top-left**) Estimated FDR demonstrates an inflection point beyond 30 ranked proteins. (**Top-right**) Nonlinear fitting reveals a rapid cumulative reverse increase. (**Bottom-left**) ROC plot shows stable classification with minimal false positives. (**Bottom-right**) Reported vs. estimated FDR highlights divergence at lower confidence levels.

*P. americana*-derived exosomes were analyzed to better understand the protein content of the exosomes and the potential role of these proteins. seventy-nine proteins were identified using LC-MS/MS; the most abundant between plant exosomes are represented in Table 1 and Figure 4.

The identified proteins (n = 79) were compared with the Vesiclepedia and Exocarta databases. This revealed that many of the identified proteins have been identified in mammalian exosomes, like glyceraldehyde-3-phosphate dehydrogenase (GAPDH), tubulin, actin, fructose-bisphosphate aldolase, alcohol dehydrogenase, and elongation factors; also, ATP synthase has been recorded in many studies in different types of exosomes from plant and animal cells.

Proteomic analysis revealed the presence of exosome protein markers such as glyceraldehyde-3-phosphate dehydrogenase (GAPDH), tubulin, and actin. Many identified proteins are involved in plant defense against stressors, like defensin, endochitinase, ethylene receptor, and 9-cis-epoxy carotenoid dioxygenase.

**Figure 5:**
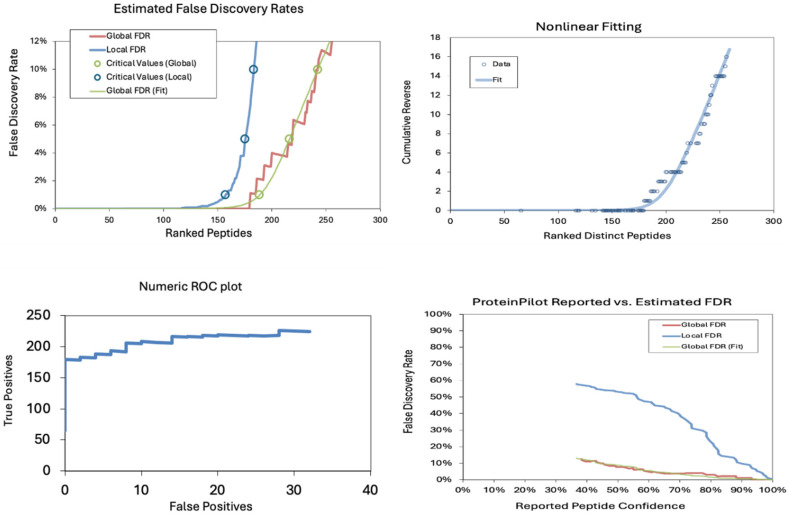
False discovery rate (FDR) analysis of identified peptides. (**Top-left**) The estimated FDR plot reveals a sharp increase in false discoveries beyond 150 ranked peptides. (**Top-right**) Nonlinear fitting indicates a rapid confidence drop past 200 peptides. (**Bottom-left**) ROC plot demonstrates a stable actual positive rate despite increasing false positives. (**Bottom-right**) Reported vs. estimated FDR comparison underscores the importance of confidence thresholds in peptide identification.

The false discovery rates (FDRs) in a proteomics dataset at the peptide level were also analyzed. The estimated false discovery rates demonstrate a sharp increase in local FDR beyond ~150 ranked peptides, indicating a critical threshold for confident identifications. The nonlinear fitting plot shows a steep rise in cumulative reverse values beyond 200 peptides, suggesting a rapid loss of confidence in peptide identifications. The Numeric ROC Plot indicates a relatively stable actual positive rate with a gradual increase in false positives, reflecting an acceptable classification performance. In contrast, the Protein Pilot Reported vs. Estimated FDR plot shows a divergence between reported and estimated FDR, with local FDR rising significantly at lower confidence levels, highlighting the importance of stringent filtering in peptide identification (Table 2 and Table 3 and Figure 5).

### 2.3. Functional Enrichment Analysis of the Identified Proteins

A KEGG and GO classification system-based bioinformatic enrichment study was carried out using ShinyGo to clarify the biological roles reflected in the proteomic profile in avocado’s extracellular vesicles. In the KEGG database analysis, identified proteins exhibited enrichment in the pathways linked with photosynthesis, such as carbon fixation, photosynthesis, respiration processes like glycolysis, and the last category, secondary metabolism.

Within the GO analysis, the identified proteins enriched terms associated with transport processes like protein import to chloroplast stroma, energy-coupled proton transport, and proton transmembrane transport, in addition to metabolic processes like ATP synthesis, purine nucleoside triphosphate biosynthetic process, oxidative phosphorylation, and carbohydrate catabolic processes; these proteins belong to the following cellular components: YCF2/FtSH complex, protein transporting ATP synthase complex, proton-transporting ATP synthase complex, mitochondrial proton-transporting ATP synthase complex, ATPase complex, mitochondrial membrane, chloroplast thylakoid membrane, external encapsulating structure. Generally, they belong to different components, such as the mitochondria, plastids, cell wall, extracellular encapsulating structures, and cytosols.

### 2.4. Biological Function

**Figure 6:**
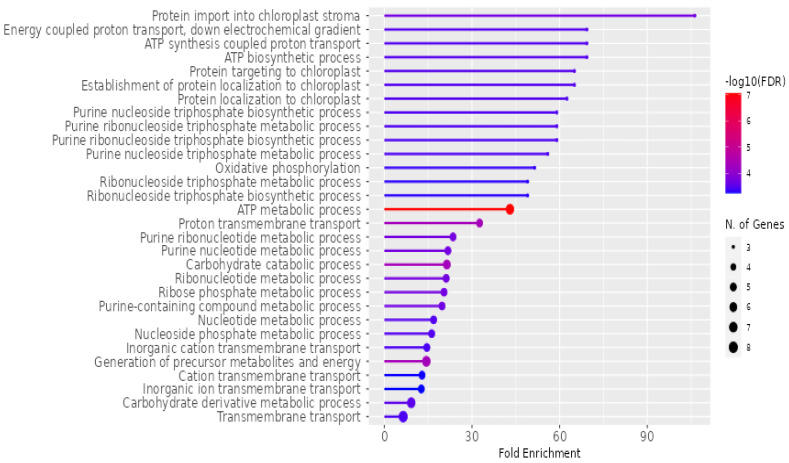
Enrichment of biological functions in *P. americana*-derived exosomes. The figure illustrates the fold enrichment (*x*-axis) and statistical significance (−log10(FDR), color gradient) of various molecular functions associated with exosomes isolated from *Persea americana*. Dot size indicates the number of genes associated with each function. Key enriched functions include transport, energy metabolism, and nucleotide metabolism, highlighting the potential roles of these exosomes in cellular processes.

The detailed analysis of the biological functions associated with *P. americana*-derived exosomes is represented in Figure 6. The horizontal axis, “Fold Enrichment”, quantifies how much more prevalent a specific function is within the exosome sample than a baseline. The color gradient of the bars signifies the statistical significance, with red hues indicating higher significance, represented as −log10(FDR). Furthermore, the size of the circles at the end of each bar corresponds to the number of genes contributing to each function, with larger circles representing a greater number of genes. Prominent biological functions highlighted include “Protein import into chloroplast stroma”, various ATP-related processes, and transmembrane transport activities. Additionally, the analysis reveals significant enrichment in metabolic pathways involving purines, nucleotides, and carbohydrates.

### 2.5. Cell Component

**Figure 7:**
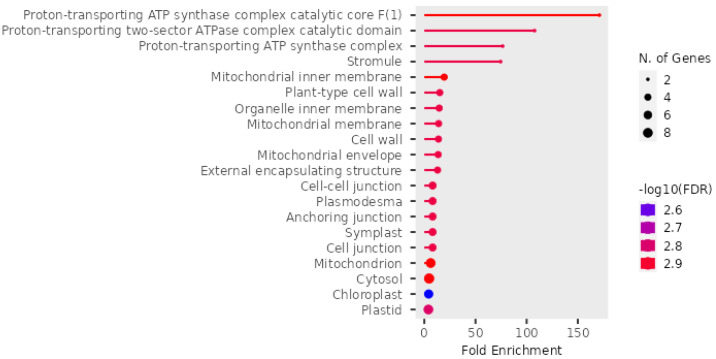
Enrichment of cell component in *P. americana*-derived exosomes. The figure depicts the fold enrichment (*x*-axis) and statistical significance (−log10(FDR), color gradient) of various biological functions associated with exosomes isolated from *Persea americana*. Dot size indicates the number of genes associated with each function. Key enriched functions include ATP synthase components, cellular localization terms, and cell junction-related functions, highlighting the potential roles of these exosomes in energy metabolism, intracellular trafficking, and cell communication.

The enrichment of various cell components associated with *P. americana*-derived exosomes is represented in Figure 7. The *x*-axis, labeled “Fold Enrichment”, quantifies how much more frequent a particular function is in the exosome sample compared to the expected frequency, providing insight into the functional relevance of these exosomes. The statistical significance of the enrichment is represented by a color gradient, where the bars are shaded according to the negative logarithm (base 10) of the false discovery rate (−log10(FDR)). Redder colors indicate higher significance, corresponding to lower FDR values, which underscores the reliability of the observed enrichments. Additionally, the size of the dots at the end of each bar corresponds to the number of genes associated with each biological function, with larger dots indicating a greater number of contributing genes. Key functions highlighted in the figure include components of the “Proton-transporting ATP synthase complex”, various cellular compartments such as mitochondria, chloroplasts, and plastids, as well as terms related to cell junctions. A significant portion of the enriched functions pertains to cellular localization and organelle components, suggesting that these exosomes play a role in intracellular trafficking and organelle-related processes. This comprehensive analysis provides valuable insights into the functional roles of avocado-derived exosomes and their potential biological significance.

### 2.6. Molecular Function

**Figure 8:**
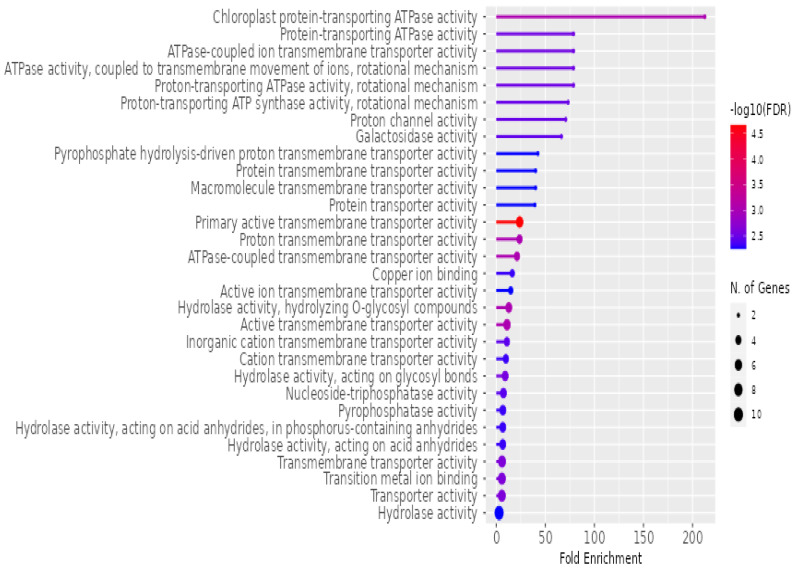
Enrichment of molecular functions in *P. americana*-derived exosomes. The figure illustrates the fold enrichment (*x*-axis) and statistical significance (−log10(FDR), color gradient) of various molecular functions associated with exosomes isolated from *Persea americana*. Dot size indicates the number of genes associated with each function. Key enriched functions include ATPase activities, various transport activities, and hydrolase activities, highlighting the potential roles of these exosomes in energy metabolism and molecular trafficking.

The enrichment of various molecular functions associated with *P. americana*-derived exosomes, as analyzed using ShinyGO v0.741 for Gene Ontology Enrichment Analysis, is presented in Figure 8. The *x*-axis, labeled “Fold Enrichment”, quantifies how much more frequent a particular molecular function is in the exosome sample compared to the expected frequency, highlighting the functional relevance of these exosomes. The statistical significance of the enrichment is depicted by a color gradient, where the bars are shaded according to the negative logarithm (base 10) of the false discovery rate (−log10(FDR)), with redder colors indicating higher significance (lower FDR). The size of the dots at the end of each bar corresponds to the number of genes associated with each molecular function, with larger dots representing a greater number of contributing genes. Key molecular functions highlighted in the figure include various ATPase activities, particularly proton-transporting ATPase, transporter activities (ion, protein, and macromolecule transport), and hydrolase activities. A significant portion of the enriched functions is related to transport processes, suggesting that these exosomes play a crucial role in the movement of ions, proteins, and other molecules across membranes, underscoring their potential involvement in cellular communication and molecular trafficking.

### 2.7. KEGG and STRING

**Figure 9:**
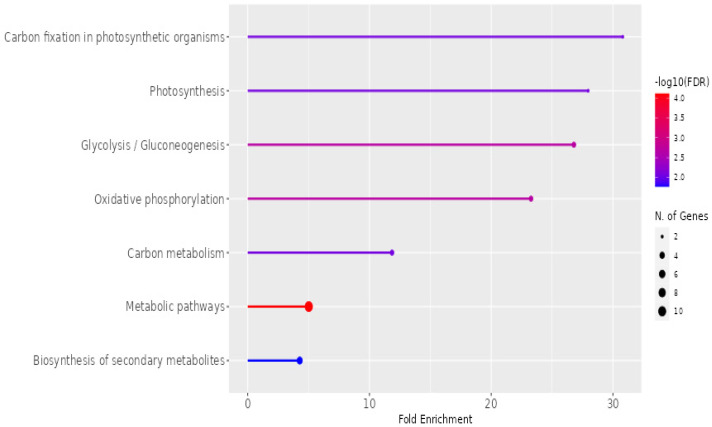
KEGG pathway enrichment in *P. americana*-derived exosomes. This figure illustrates the fold enrichment and statistical significance of various metabolic pathways associated with exosomes from *Persea americana*. The high enrichment of photosynthetic and glycolysis pathways suggests a significant role for these exosomes in core metabolic functions.

The KEGG pathway enrichment analysis of exosomes isolated from *P. americana*, showcasing the fold enrichment and statistical significance of various metabolic pathways, is presented in Figure 9. The *x*-axis represents “Fold Enrichment”, indicating the overrepresentation of specific pathways in the exosome sample. At the same time, the color gradient of the bars signifies the statistical significance, denoted by −log10(FDR), with redder colors indicating higher significance. The size of the dots at the end of each bar corresponds to the number of genes associated with each pathway. Notably, “Carbon fixation in photosynthetic organisms”, “Photosynthesis”, and “Glycolysis/Gluconeogenesis” exhibit the highest enrichment and statistical significance, suggesting a strong involvement of these exosomes in fundamental metabolic processes. Other enriched pathways include “Oxidative phosphorylation”, “Carbon metabolism”, “Metabolic pathways”, and “Biosynthesis of secondary metabolites”, highlighting the diverse metabolic roles potentially played by these exosomes.

**Figure 10:**
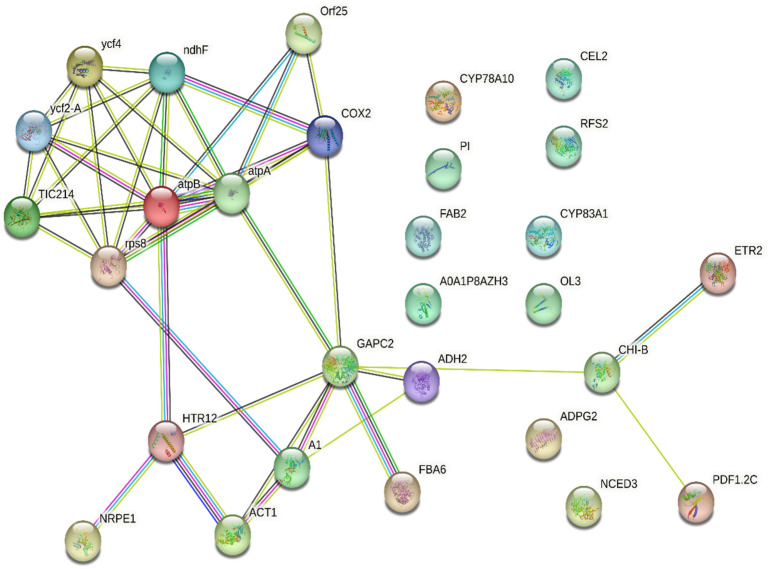
Comprehensive protein–protein interaction network of DEPs in *Persea americana*-derived exosomes. This figure illustrates the intricate network of interacting proteins within avocado exosomes, highlighting key hub proteins and functional modules that may contribute to their diverse biological activities.

To elucidate the relation between the identified proteins, they were uploaded into the STRING online database for protein–protein interaction analysis, and the resultant networks are illustrated in Figure 10. The study revealed the relation between 18 protein nodes representing Photosystem I assembly protein Ycf4, Protein Ycf2, Protein TIC 214, 30S ribosomal protein S8, ATP synthase subunit beta, ATP synthase subunit alpha, Cytochrome c oxidase subunit 2, NAD(P)H-quinone oxidoreductase subunit 5, ATP synthase protein MI25, DNA-directed RNA polymerase V subunit 1, Histone H3-like centromeric protein HTR12, Actin-1, Elongation factor 1-alpha 1, Glyceraldehyde-3-phosphate dehydrogenase, Fructose-bisphosphate aldolase 6, Alcohol dehydrogenase class-3, Basic endochitinase B, Defensin-like protein 17, and Ethylene receptor 2.

The comprehensive protein–protein interaction (PPI) network of differentially expressed proteins (DEPs) identified in exosomes isolated from *P. americana* (avocado) was represented. This network visualizes the complex relationships between these proteins, where nodes represent individual proteins and edges indicate interactions. The network highlights interacting protein clusters, suggesting functional modules within the exosomes. Several proteins like COX2, GAPC2, and CHI-B appear to serve as central hubs, indicating their potential roles in coordinating exosomal functions. The presence of proteins like atpA and atpB suggests involvement in energy-related processes, while others, such as CYP78A10 and CYP83A1, hint at roles in metabolic pathways. The intricate connectivity of this network underscores the complexity of exosomal cargo and its potential to influence various cellular processes.

### 2.8. Encapsulation Efficiency

In *P. americana*-derived exosomes, the encapsulation efficiencies of DOX were 18 ± 0.22%. Novel techniques, including sonication and extrusion-assisted active loading, may be employed to achieve more effective loading. The release pattern of DOX showed a burst release during the initial hours of incubation, followed by a steady release until the experiment’s termination at 44.4 ± 4.4% at 24 h.

### 2.9. MTT Assay

**Figure 11:**
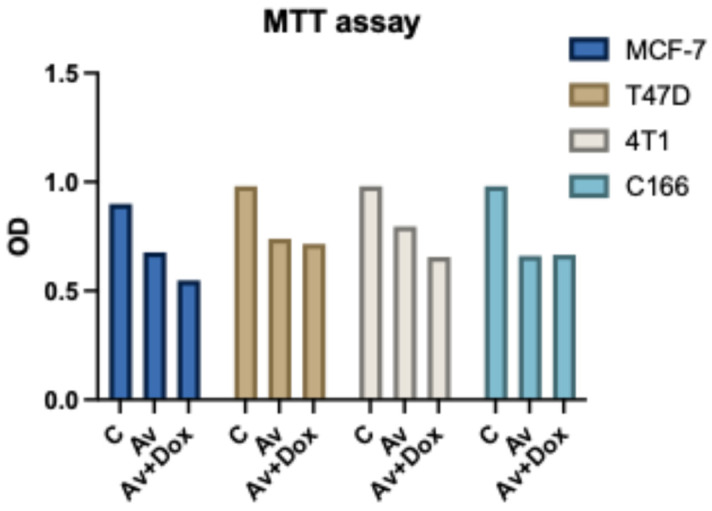
MTT assay showing the viability of MCF-7, T47D, 4T1, and C166 cell lines after treatment with Av and Av+DOX. Av treatment reduces cell viability, and the combination of Av+DOX significantly enhances cytotoxicity, particularly in cancer cell lines. Error bars represent standard deviations from triplicate experiments.

The MTT assay results indicate that treatment with *P. americana*-derived exosomes reduces cell viability in T47D, MCF-7, 4T1, and C166 cell lines compared to the control. Combining Av with DOX (Av+DOX) further decreases viability, particularly in the cancer cell lines, suggesting enhanced cytotoxicity. Notably, the viability reduction in C166 is less pronounced, indicating potential cell-type-specific effects (Figure 11).

### 2.10. Gene Expression

**Figure 12:**
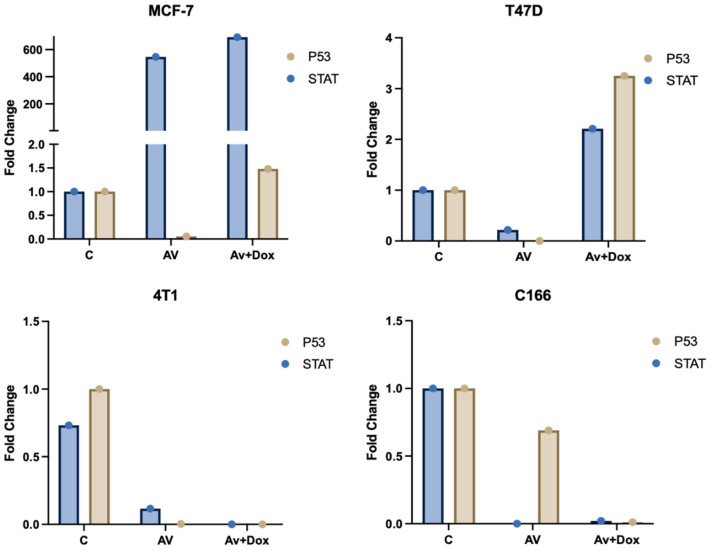
Effect of *Persea americana*-derived exosomes (Av) and DOX on *TP53* and *STAT* gene expression. Relative fold change in *TP53* (p53, yellow bars) and *STAT* (blue bars) gene expression in MCF-7, T47D, 4T1, and C166 cell lines after treatment with Av alone or Av in combination with DOX (Av+Dox), compared to untreated control (C) cells (normalized to 1). Data represent mean fold change.

Differential expression patterns of *TP53* (p53) and *STAT* genes were observed across the MCF-7, T47D, 4T1, and C166 cell lines following treatments. Treatment with *P. americana*-derived exosomes (AV) alone resulted in distinct, cell-specific effects: it caused a dramatic upregulation of *STAT* (approx. 480-fold) coupled with significant *p53* downregulation in MCF-7 cells, while strongly suppressing both genes (especially *STAT*, near zero) in 4T1 and C166 lines, and inducing only minor changes in T47D. Adding DOX to the exosomes (Av+Dox) produced further varied outcomes: it maintained the high *STAT* expression in MCF-7 while partially restoring *p53* levels and synergistically induced strong upregulation of both *p53* (~3.3-fold) and *STAT* (~2.2-fold) in T47D cells, but failed to reverse the gene suppression seen in the 4T1 and C166 lines compared to Av treatment alone (Figure 12).

## 3. Discussion

Plant-derived extracellular vesicles (EVs), particularly exosome-like nanoparticles, have emerged as a promising therapeutic tool in cancer treatment. Multiple studies have demonstrated their intrinsic anticancer properties, which are further enhanced when loaded with chemotherapeutic agents such as doxorubicin (DOX). Exosomal drug delivery systems integrate the advantages of nanotechnology and targeted drug transport, overcoming numerous biological barriers to improve drug efficacy and reduce toxicity. Unlike synthetic nanoparticles, which may elicit toxicity and immune rejection, plant-derived EVs exhibit superior biocompatibility and safety profiles, making them highly attractive candidates for drug delivery applications [34,35].

Plant-derived extracellular vesicles differ significantly from mammalian-derived EVs in their lipid composition and bioactive cargo. However, emerging evidence supports their ability to interact with and be internalized by mammalian cells, facilitating cross-kingdom communication [36]. This interaction underscores the potential of plant-derived exosomes as a novel platform for targeted drug delivery. In this study, *P. americana* (avocado) exosomes demonstrated a strong capacity to transport therapeutic molecules, in alignment with prior research on other plant-derived vesicles such as those from ginger and grapes [23,24].

Avocado has been reported to exhibit significant anticancer activity against various cancer cell lines. According to [37], methanolic extracts from avocado seeds induced apoptosis in MDA-MB-231 breast cancer cells. Furthermore, avocado fruit extracts have shown apoptotic and cell-cycle arrest properties against different cancer models. This inherent cytotoxicity, combined with exosomal drug delivery, reinforces the potential of avocado-derived exosomes as a robust anticancer therapy.

Proteomic characterization of avocado-derived exosomes identified 79 proteins, many of which are well-established markers of plant exosomes, including glyceraldehyde-3-phosphate dehydrogenase (GAPDH), tubulin, actin, and elongation factor 1α (EF-1α). Interestingly, many of these proteins have also been identified in mammalian extracellular vesicles, as documented in the Vesiclepedia database. These shared components suggest functional similarities between plant and mammalian exosomes in vesicle biogenesis, cargo sorting, and intercellular communication.

Among the most frequently detected proteins, GAPDH plays a crucial role in EV assembly and release. It is the third most abundant protein in EVs globally, recorded 377 times in Vesiclepedia, and ranks fourth in exosomes according to ExoCarta. In addition to its metabolic function in glycolysis, GAPDH contributes to exosome-mediated signal transduction, immune modulation, and therapeutic potential.

Another key enzyme identified in *P. americana*-derived exosomes was fructose-bisphosphate aldolase, which has been detected 69 times in exosomes across different organisms). This enzyme is not only involved in glycolysis, converting fructose-1,6-diphosphate into GADPH [38], but also plays a pivotal role in actin polymerization and ATP synthesis, processes that are fundamental for vesicle formation and cellular energy metabolism [39].

Elongation factor 1α (EF-1α), another highly abundant exosomal protein, was recorded 71 times in exosomes. It facilitates protein translation by ensuring accurate codon–anticodon pairing and has been widely documented in EVs due to its role in mRNA stabilization and ribosomal function [40]. Additionally, histone H3, a chromatin-associated protein frequently identified in mammalian small extracellular vesicles (sEVs), was also detected in *P. americana*-derived exosomes, further supporting their structural and functional similarity to mammalian exosomes [31,41].

Alcohol dehydrogenase, among the top 100 most frequently detected proteins in vesicles, was also identified in *P. americana*-derived exosomes. This enzyme has been previously found in liver-derived exosomes and plays a role in detoxification, oxidative stress regulation, and plant defense mechanisms [42]. Additional ribosomal proteins, such as chloroplastic 30S ribosomal protein and L2, were among the top 50 most abundant proteins in ectosomes and have been extensively documented in Vesiclepedia.

Furthermore, chitinase PR3 and PR2, proteins widely recognized as plant exosomal markers, were identified in this study. These enzymes have been isolated from various fruit-derived EVs and are involved in pathogen defense [43]. The detection of conserved oligomeric Golgi complex subunit 7, a vesicle assembly complex essential for protein sorting and lipid glycosylation, suggests that plant exosomes, like mammalian exosomes, originate from the Golgi apparatus-endosomal pathway [44].

KEGG pathway enrichment analysis revealed that the identified proteins were significantly associated with photosynthesis, carbon fixation, glycolysis, and secondary metabolism, providing valuable insights into the biological functions of plant-derived EVs. Gene Ontology (GO) analysis also classified the identified proteins into diverse cellular components, including mitochondria, plastids, the cell wall, extracellular encapsulating structures, cell junctions, and the cytosol.

Cell wall remodeling enzymes, such as endoglucanase, support the hypothesis that plant exosomes participate in structural modifications and cross-kingdom interactions. Previous studies have reported similar enzymes in EVs isolated from various plant organs. Other identified defense-related proteins, including defensin, endochitinase, ethylene receptor, and 9-cis-epoxy carotenoid dioxygenase, suggest that *P. americana*-derived exosomes may exert additional immunomodulatory effects beyond drug delivery.

Notably, glutathione S-transferase (GST), a key enzyme in oxidative stress response, was detected in *P. americana*-derived exosomes. This enzyme has also been reported in exosomes from *Aloe vera* plants, further supporting the conservation of stress-related mechanisms in plant-derived EVs [45]. Additionally, proteins involved in mitochondrial respiration, such as cytochrome c oxidase, were identified, aligning with findings from mammalian EVs where oxidases contribute to vesicle metabolism and cellular respiration [46].

In this study, DOX was successfully loaded into avocado exosomes using a simple, efficient procedure. The resulting DOX-loaded exosomes demonstrated potent anticancer effects, reinforcing the hypothesis that plant-derived EVs are effective drug carriers.

Our findings align with previous studies showing that exosome-loaded therapies outperform free drugs or synthetic carriers in therapeutic efficiency. Kim et al. [47] reported that exosomal DOX circumvents multidrug resistance (MDR) by evading efflux transporters, improving intracellular drug retention. Similarly, exosomal carriers have enhanced drug bioavailability and stability compared to conventional nanocarriers like liposomes [48].

Despite their widespread application in drug delivery, synthetic nanoparticles have multiple limitations, including cytotoxicity, immune activation, and lower biocompatibility [34]. In contrast, plant exosomes, derived from natural membranes, retain integrins, proteoglycans, and lectins, facilitating specific cell targeting and uptake [49]. Moreover, plant exosomes have demonstrated superior safety and efficacy in drug delivery applications [35].

Additionally, avocado exosomes naturally contain bioactive molecules with anticancer potential. Defensin, a potent antimicrobial peptide, has been detected in *P. americana*-derived exosomes and mammalian exosomes. Studies have shown that defensin disrupts breast cancer cell growth and metastasis by interfering with exosomal CD63 and CD9 recruitment [50,51]. NADPH quinone oxidase, another identified protein, stabilizes p53 through redox regulation, contributing to its antitumor activity [52].

## 4. Materials and Methods

### 4.1. Isolation and Purification of P. americana-Derived Exosome

Exosomes were isolated from *P. americana* according to standard protocols. Briefly, 500 g of fresh *P. americana* fruit was brought from the local market, washed thoroughly, and homogenized using a juicer at 18,000 rpm for 3 min. The juice was filtered using a nylon mesh (pore size 200 μm). The filtrate was centrifuged at low speed, 2000× *g* for 10 min, then 6000× *g* for 20 min at 4 °C, and at medium speed, 10,000× *g* for 45 min. The PEG6000 (Sigma Aldrich, St. Louis, MO, USA) was added to the obtained supernatant to achieve a final concentration of 15%, and then, the mixture was preserved in the refrigerator at 4 °C. The mixture was centrifuged at low speed (8000× *g* for 45 min) to purify avocado exosomes. The centrifuge tube was inverted upside down to eliminate excess supernatant, and sterile water was added to adjust the concentration of exosomes to 0.5 mg/μL, weight/volume. The sample was dialyzed overnight against milli-Q water using a dialysis membrane (Himedia, Thane, India) with a pore size of 10 kDa.

### 4.2. Characterization of P. americana Exosome

Characterization of avocado exosomes was carried out according to the method of Théry et al. [31] with some modifications. Briefly, 5 μL of the prepared exosomes were fixed in 2% paraformaldehyde (PFA) and transferred to carbon-coated electron microscopy grids. Then, fixed exosomes were left for 20 min at room temperature in a dry environment. Exosomes were fixed in 1% glutaraldehyde for 5 min, then washed eight times with distilled water, each wash lasting for 2 min, and contrasted in 2% uranyl-oxalate for 5 min and embedded in a mixture of 4% uranyl-acetate and 2% methylcellulose for 10 min on ice. The exosomes were examined and photographed using a JEOL GEM-1010 transmission electron microscope (JEOL, Tokyo, Japan) with a 70 kV accelerating voltage to determine the particle size and the shape.

### 4.3. Size Distribution of P. americana Exosome

The size distribution of *P. americana* exosomes was determined by DLS. The exosome preparation (10 μL, n = 12) was diluted with sterilized Milli-Q-water (90 μL). Then, 50 μL was transferred to a cuvette with a 10 mm path length. The analysis was performed using a Nano Zetasizer (Malvern Instruments, Malvern, UK) at room temperature using 633 nm and recording the backscattered light at an angle of 173°. The recording lasted for 150 s. Three measurements were carried out for each sample. Dispersion Technology Software v.5.10 (Malvern Instruments) was employed to convert DLS signal intensity to size distribution. The peak maximum of the Gaussian function was used to measure exosome diameter (nm). The intensity-based distribution was converted to a volume.

### 4.4. Protein Extraction

The total proteins were isolated from extracellular lipid nanovesicles (ELNs), as detailed below. About 100 μL of 8 M Urea (500 mM Tris pH 8.5) was initially combined with 50 μL of ELNs. The solution was centrifuged at 10,000 rpm for 30 min. The supernatant was subsequently utilized for downstream procedures, and the bicinchoninic acid assay (BCA test) was conducted to quantify protein content.

### 4.5. Protein Denaturation and Digestion

After determining protein content, the isolated protein was subjected to denaturation: about 2 μL of DTT (200 mM) was first added to the protein solution, followed by vortex mixing. The mixture was then left for 45 min at room temperature in the dark after adding 2 μL of 1 M iodoacetamide. The solution, consisting of 102 μL of 100 mM Tris at pH 8.5 and 6 μL of Trypsin containing 1 μg of porcine enzyme, was then placed in a shaker incubator for 12 h at 37 °C. The pH of the solution was lowered to 2 using 6 μL of 100% formic acid, and the Eppendorf tube was inverted on a paper towel for 30 min. The concentration of the peptide was assessed utilizing the bicinchoninic acid assay.

An amount of 10 microliters containing 1 μg of peptides was injected into a trap column, ChromXP C18CL (5 μm, 10 × 0.5 mm). The material was eluted into a 3 μm C18 analytical column, ChromXP C18CL (120 Å, 150 × 0.3 mm). The mobile phase consisted of A: LC-MS water containing 0.1% fluoroacetic acid, and B: acetonitrile with 0.1% fluoroacetic acid. The time-dependent gradient composition of the two components, A and B, reflects a controlled solvent transition, likely used in liquid chromatography. Initially, at 0 min, the composition is 97% A and 3% B, maintaining a high concentration of A. Over time, component B gradually increases, reaching 30% at 38 min and 40% at 43 min, marking a transition phase. A significant shift occurs at 45 min, where A drops to 20% and B rises to 80%, indicating a crucial elution phase. This composition remains stable at 48 min, suggesting a potential isocratic hold before the system returns to its initial state at 49 min (97% A, 3% B), remaining unchanged at 57 min and completing the re-equilibration process. This pattern is characteristic of gradient elution in analytical and pharmaceutical chemistry, ensuring the effective separation of compounds by modulating solvent composition for optimal resolution and identification of molecular components.

The flow rate was 5 μL per min. Raw MS data from the TripleTOFTM 5600+ (AB SCIEX Foster City, CA, USA) were analyzed using Protein Pilot (version 5.0.1.0, 4895) and the Paragon Algorithm (version 5.0.1.0, 4874). The utilized databases were Uniprot for the organism *P. americana*, encompassing the Swiss-Prot and TrEMBL databases comprising 534 proteins.

### 4.6. Preparation of DOX-Loaded Exosomes

The DOX–exosome formulation was prepared by mixing the DOX solution with the exosome solution. Then, the former solution was incubated at 37 °C for 1 h. After that, the mixture was centrifuged at 10,000× *g* for 10 min to eliminate the untapped drug, and then, the exosomal pellets were washed with water two times and centrifuged at the same speed. The loaded exosomes were sterilized by passing the exosome solution through a 0.22 µm syringe; finally, the loaded exosomes were stored at −80 °C until use.

### 4.7. Measurement of Doxorubicin in Exosomes

The presence of DOX in *P. americana* exosomes was tested by HPLC. The complete structure of the separated exosomes was preserved using a lysis buffer containing 10% Triton X-100 in methanol, and the concentration of DOX was later quantified in the solution. The mobile phase included acetonitrile and water (pH = 3) in a 70:30 ratio, with a flow rate of 1 mL per minute. A UV detector analyzed the absorption spectrum at 233 nm. Four solutions with serial dilutions of DOX (5.2, 5, 10, and 20 ppm) were produced and introduced into the device to construct a standard curve.

### 4.8. Encapsulation Efficiency and Drug Release

As previously mentioned [13], a 1 mg/mL DOX-EX solution was centrifuged for 30 min at 16,000× *g* to ascertain encapsulation efficiency (EE). The supernatants were moved to eliminate unbound DOX from the prepared mixture, and the pellet underwent two ethanol washes. Via the UV spectrum, the amount of DOX in each of the resultant supernatants was measured at an absorbance of 490 nm. The DOX quantity was calculated using a reference curve with known DOX concentrations. The following formula was used to calculate encapsulation efficiency:

(EE%) = (Wt/Wi) × 100%

where Wt is the total amount of drug in the nanovesicle suspension, and Wi is the total quantity of drug added initially during preparation.

By gently stirring the nanoparticles (NPs) in phosphate-buffered saline (pH 7.4) at 37 °C, in vitro release was assessed. Samples were taken at various intervals (1, 2, 4, 8, and 24 h), and the amount of DOX released from the NPs was measured using the methodology previously mentioned.

### 4.9. Cell Culture

MCF-7, T47D, and 4T1 human breast cancer cells and C166 cells, mouse endothelial cell line, as normal cells were obtained from the Holding Company for Biological Products (Cairo, Egypt). Cells were maintained in DMEM (Dulbecco’s Modified Eagle Medium) supplemented with 10% fetal bovine serum (FBS) and 1% penicillin-streptomycin. Cells were cultured in a humidified incubator at 37 °C with 5% CO_2_ until reaching 80–90% confluence, after which they were passaged or harvested for experiments. C166 cells were cultured under similar conditions but were routinely split at 70–80% confluence to avoid overgrowth. All cells were treated with either Av (*P. americana*-derived exosomes) (10 μg/mL), DOX (2 μg/mL), or a combination of Av and DOX (10 μg/mL) for 24 h.

### 4.10. MTT Assay

The tetrazolium-based colorimetric (MTT) assay was used to assess the cytotoxicity of *P. americana*-derived exosomes, DOX, and DOX-loaded exosomes. The MCF-7, T47D, 4T1, and C166 cells were seeded at a density of 5 × 10^3^ per well in 96-well plates and then incubated for 24 h at 37 °C in an incubator with 5% CO_2_ in either vehicle (DMSO) or appropriate quantities of exosomes or DOX and DOX-loaded exosomes. Subsequently, 100 μL of new media devoid of serum and 10 μL of MTT (5 mg/mL; Sangon Biotech, Shanghai, China) solution were added to the medium, and the mixture was incubated for an extra 4 h at 37 °C with 5% CO_2_. The medium was then removed, and 100 μL of DMSO was added. The microplate reader was utilized to measure the absorbance at 490 nm (Bio-Rad Laboratories Inc., Hercules, CA, USA). The absorbance ratio between the treatment and control group cells was used to determine the cytotoxicity of used treatments.

### 4.11. RNA Isolation

Total RNA was extracted from MCF-7, T47D, 4T1, and C166 cells using the TRIzol reagent (Invitrogen, Carlsbad, CA, USA). Following trypsinization, cells were centrifuged at 300× *g* for 5 min. The supernatant was discarded, and the pellet was resuspended in 1 mL of TRIzol reagent. After a 5 min incubation at room temperature, 200 µL of chloroform was added, and the mixture was vigorously shaken. After a 2–3 min incubation, samples were centrifuged at 12,000× *g* for 15 min at 4 °C, and the aqueous phase was collected for RNA precipitation using isopropanol. The RNA pellet was washed with 70% ethanol, air-dried, and resuspended in RNase-free water. RNA concentration and purity were evaluated using a NanoDrop spectrophotometer (Thermo Fisher Scientific, Waltham, MA, USA).

### 4.12. cDNA Synthesis

Complementary DNA (cDNA) was synthesized from 1 µg of total RNA from each cell line using the High-Capacity Reverse Transcription Kit (Applied Biosystems, Foster City, CA, USA) following the manufacturer’s instructions. The reaction mixture was incubated at 37 °C for 60 min and heat-inactivated at 95 °C for 5 min. The cDNA was stored at −20 °C for subsequent quantitative PCR analysis.

### 4.13. Quantitative RT-PCR

Quantitative PCR was conducted to quantify the expression levels of TP53 and STAT3, utilizing specific primers for both genes: for *TP53*, the forward primer was 5′-AGTACCCAGAGGACCAGGAC-3′ and the reverse primer was 5′-CAGGTTTGCTTTCCAGGACA-3′; for *STAT3*, the forward primer was 5′-GCTGACCAGTAGCCACAGG-3′ and the reverse primer was 5′-CTGCTGCTTTGGTGATGCTG-3′. The qPCR reactions were performed using SYBR Green PCR Master Mix (Applied Biosystems, Foster City, CA, USA) in a total volume of 20 µL, where each reaction contained 1 µL of cDNA template, 200 nM of each primer, and 10 µL of SYBR Green Master Mix. Reactions were run in triplicate on a Real-Time PCR System (Applied Biosystems) with thermal cycling conditions comprising an initial denaturation at 95 °C for 10 min, followed by 40 cycles of denaturation at 95 °C for 15 s and annealing/extension at 60 °C for 1 min. A melt curve analysis was conducted post-amplification to confirm the specificity of the products. Gene expression levels were quantified using the ΔΔCt method, with *GAPDH* as the housekeeping gene. Relative expression levels were compared across different treatments.

### 4.14. Statistical Analysis

The data were expressed as means ± standard error of the mean (SEM) using one-way analysis of variance (ANOVA) to compare the differences among means of multiple groups. *p* < 0.05 was considered a significant result.

## 5. Conclusions

This study highlights the potential of *P. americana*-derived exosomes as a promising, natural nanocarrier for drug delivery. The proteomic analysis revealed that *P. americana*-derived exosomes contain several key proteins commonly found in mammalian extracellular vesicles, including GAPDH, actin, tubulin, and elongation factor 1α, suggesting functional similarities between plant and mammalian exosomes. Additionally, identifying proteins involved in glycolysis, oxidative stress response, mitochondrial respiration, and immune modulation further supports the biological significance of *P. americana*-derived exosomes in cellular communication and defense mechanisms. Doxorubicin (DOX) was successfully loaded into *P. americana*-derived exosomes, and the DOX-loaded exosomes exhibited enhanced anticancer efficacy, likely due to their superior biocompatibility, cellular uptake, and stability compared to synthetic nanoparticles. Furthermore, *P. americana*-derived exosomes naturally contain bioactive molecules such as defensin and NADPH quinone oxidase, which have demonstrated anticancer properties, reinforcing their therapeutic potential. Unlike synthetic nanocarriers, plant-derived exosomes offer low toxicity, immune evasion, and targeted delivery capabilities, making them an attractive alternative for drug delivery applications. This study proves that plant-derived exosomes, particularly from *Persea americana*, can serve as efficient, safe, and biocompatible drug delivery systems. Future research should focus on in vivo validation, mechanistic studies, and clinical translation to fully exploit their potential in cancer therapy and other biomedical applications.

## Figures and Tables

**Table 1:** Proteins identified at critical false discovery rates.

Number of Proteins Detected			
Critical FDR	Local FDR	Global FDR	Global FDR from Fit
1.0%	31	30	31
5.0%	31	30	32
10.0%	31	32	34

**Table 2:** Peptides identified at critical false discovery rates.

Number of Peptides Identified			
Critical FDR	Local FDR	Global FDR	Global FDR from Fit
1.0%	157	179	188
5.0%	175	218	216
10.0%	183	256	242

**Table 3:** Spectra identified at critical false discovery rates.

Number of Spectra Identified			
Critical FDR	Local FDR	Global FDR	Global FDR from Fit
1.0%	301	338	347
5.0%	324	389	392
10.0%	334	466	442

## Data Availability

Data is contained within the article.

## Abbreviations

ANOVA: Analysis of variance; ATP: adenosine triphosphate; BCA: bicinchoninic acid; DLS: dynamic light scattering; DOX: doxorubicin; EE: encapsulation efficiency; EF-1α: elongation factor 1 alpha; ELNs: extracellular-like nanoparticles; EVs: extracellular vesicles; ExoCarta: exosomal database; FDR: false discovery rate; GAPDH: Glyceraldehyde-3-Phosphate Dehydrogenase; GO: Gene Ontology; GST: glutathione S-transferase; HPLC: High-Performance Liquid Chromatography; KEGG: Kyoto Encyclopedia of Genes and Genomes; LC-MS/MS: Liquid Chromatography–Tandem Mass Spectrometry; MCF-7: Michigan Cancer Foundation-7 (human breast cancer cell line); MDA-MB-231: human breast cancer cell line; MDR: multidrug resistance; miRNA: microRNA; MPS: mononuclear phagocytic system; mRNA: messenger RNA; MTT: 3-(4,5-Dimethylthiazol-2-yl)-2,5-diphenyltetrazolium Bromide; PEs: plant-derived exosomes; PPI: protein–protein interaction; sEVs: small extracellular vesicles; SEM: standard error of the mean; STAT3: Signal Transducer and Activator of Transcription 3; STRING: Search Tool for the Retrieval of Interacting Genes/Proteins; TEM: transmission electron microscopy; TIC: Translocon at the Inner Envelope Membrane of Chloroplasts; UV: ultraviolet.
